# The role of miR-485-5p/NUDT1 axis in gastric cancer

**DOI:** 10.1186/s12935-017-0462-2

**Published:** 2017-10-17

**Authors:** Jingjing Duan, Haiyang Zhang, Shuang Li, Xinyi Wang, Haiou Yang, Shunchang Jiao, Yi Ba

**Affiliations:** 1 Medical College, Nankai University, Weijin Road 94, Tianjin, 300071 China; 2 Department of Gastrointestinal Oncology, Tianjin Medical University Cancer Institute and Hospital, National Clinical Research Center for Cancer, Tianjin Key Laboratory of Cancer Prevention and Therapy, Tianjin’s Clinical Research Center for Cancer, Huan hu xi Road 18, Tianjin, 300060 China; 3 Department of Oncology, Chinese PLA General Hospital, Fuxing Road 28, Beijing, 100853 China

**Keywords:** miR-485-5p, NUDT1, Gastric cancer, Proliferation, Migration

## Abstract

**Background:**

Cancers can survive the oxidative conditions by upregulating nucleoside diphosphate linked moiety X-type motif 1 (NUDT1). However, the mechanisms underlying gastric carcinogenesis and the dys-regulation of NUDT1 in gastric cancer (GC) remain unknown. Our study aimed to explore the role of NUDT1 and its regulatory pathway by miR-485-5p in GC.

**Methods:**

Gastric cancer tissues and paired noncancerous tissue samples were collected, and the expression level of NUDT1 and miR-485-5p were detected. Two cohorts from The Cancer Genome Atlas (TCGA) database and another cohort from the Tianjin Medical University Cancer Institute and Hospital were further analyzed. Luciferase assays were performed, and the effects of the miR-485-5p/NUDT1 axis on GC cells and normal gastric cells were determined by subsequent experiments.

**Results:**

We found that the expression of miR-485-5p was clearly repressed in GC tissues, while NUDT1 expression level was dramatically increased. The overexpression of NUDT1 correlated closely with an increase in invasive depth and a decrease in survival in GC patients. MiR-485-5p could directly bind to the 3′UTR of NUDT1 mRNA and induce its degradation, thus down-regulate its expression. The miR-485-5p/NUDT1 axis could lead to the changes of 8-oxo-dG in GC cells. And the increased expression of NUDT1 resulting from the downregulation of miR-485-5p could accelerate cell proliferation and metastasis in GC. However, the growth and migration of normal gastric cells did not depend on the protection of NUDT1, while the overexpression of NUDT1 could promote malignant transition in normal gastric cells.

**Conclusions:**

MiR-485-5p acts as a tumor suppressor by targeting NUDT1 in GC. The miR-485-5p/NUDT1 axis is involved in the processes of cell growth and cell motility and plays a key role in the tumorigenesis of GC.

**Electronic supplementary material:**

The online version of this article (doi:10.1186/s12935-017-0462-2) contains supplementary material, which is available to authorized users.

## Background

Reactive oxygen species (ROS) are generated during regular electron transport in the mitochondria or other cellular metabolic pathways. Cellular macromolecules, including nucleic acids, proteins and lipids, are always at high risk of being oxidized by ROS and thus lead to functional or structural abnormalities. Oxidative damage to nucleic acids is the most hazardous owing to the alteration of gene information caused by base mismatch to oxidative nucleotide, such as 8-oxo-7,8-dihydro-2′-deoxyguanosine 5′-triphosphate (8-oxodGTP). Accumulation of 8-oxodGTP in genomes can result in mutagenesis or cell death, and is eventually minimized by the functions of nucleoside diphosphate linked moiety X-type motif 1 (NUDT1), also known as MutT homolog-1 (MTH1) with its activity of 8-oxo-dGTPase [1]. NUDT1 can hydrolyze 8-oxodGTP into 8-oxodGMP or 8-oxodGDP, then sanitize from the nucleotide pools [1].

With high proliferative properties and various microenvironmental factors, cancer tissues are exposed to high oxidative pressure levels and thus accumulate a high level of 8-oxo-dGTP in their nucleotide pools [2–5]. As a result, cancer cells up-regulate NUDT1 to eliminate excessive 8-oxo-dGTP [5–7], indicating that increased expression of NUDT1 in cancer cells may be detrimental for cancer patients. Overexpression of NUDT1 may have several protective functions for cancer cells by hydrolyzing 8-oxo-dGTP or other oxidized nucleotides produced by endogenous elevated ROS or by therapy-induced ROS, thus contributing to malignant phenotypes, poor prognosis and resistance to therapy in cancer patients. It has been reported that NUDT1 is overexpressed in various cancers [8, 9]. And some positive correlations have been observed between the expression levels of NUDT1 and the prognosis of cancer patients [10–12]. Recent studies also demonstrate that NUDT1 is indispensable for the oncogenic RAS-mediated transformation and proliferation in tumorigenic cells, because oncogenic RAS can induce ROS formation [13–15]. Since normal tissues are in regular cell cycles and are with low oxidative pressure, the actions of NUDT1 are unnecessary. Based on the protective properties of NUDT1 against oxidative stress in cancer cells and the nonessential role of it in normal cells, NUDT1 inhibitors have been developed as potential anti-cancer drugs [2, 16].

Gastric cancer (GC) is the fourth most common cancer and the third lethal cancer worldwide [17]. The morbidity and mortality of GC in China is much higher than others, both rank second merely after lung cancer [18]. Target therapy is a promising strategy in the cancer treatment, however, the effective target used in the treatment of GC is limited. Hence, many scholars focus on the mechanisms of GC oncogenesis to discover novel targets. It is noteworthy that GC is also in high oxidative stress, so GC cells theoretically require the overexpression of NUDT1 to survive. However, the mechanism underlying the dys-regulation of NUDT1 in cancer, particularly in GC remains unknown.

MicroRNAs (miRNAs) are significant modulators in carcinogenic pathways by specific mRNA cleavage or translational repression of target genes. Whether miRNAs play an important role in the dys-regulation of NUDT1 in GC is unidentified. The tumor suppressor role of miR-485-5p was discovered in several cancers [19–21]. A circulating non-coding RNA panel, including miR-485-5p, was reported to act as an early detection predictor of non-small cell lung cancer [22]. Down-regulation of miR-485-5p was also found in GC tissues and involved in predicting prognosis in GC patients [23, 24]. However, the molecular mechanism that miR-485-5p regulate tumorigenesis in GC remains largely unspecified. Bioinformatics tools showed that NUDT1 might be a target of miR-485-5p. Hence, this study was carried out to investigate the actual role of miR-485-5p and its relationship with the ROS scavenger NUDT1 in the oncogenesis of GC.

## Methods

### Human tissues and clinical cohorts

Fresh GC tissues and paired adjacent noncancerous tissues were obtained from patients undergoing a radical surgery at the Tianjin Medical University Cancer Institute and Hospital. Tissue fragments were immediately frozen in liquid nitrogen at the time of surgery. Formalin-fixed, paraffin-embedded (FFPE) sections of GC specimens and paired adjacent noncancerous specimens were derived from 40 GC patients with complete clinicopathological and follow-up information who underwent radical surgery from October 2009 to December 2009 at the Tianjin Medical University Cancer Institute and Hospital. Tumor tissues were histopathologically verified adenocarcinoma and noncancerous tissues were confirmed negative. All aspects of the study were approved by the Ethics Committee of Tianjin Medical University Cancer Institute and Hospital and informed consent was obtained before surgery.

A total of 407 GC patients with mRNA expression profiling and 491 GC patients with miRNA expression profiling were included from The Cancer Genome Atlas (TCGA) database.

### Cell lines and culture

Human gastric cancer cell line SGC7901 and MGC803, normal gastric cell line GES-1 and embryo kidney epithelial cell line HEK-293T were cultured following instructions.

### The miRNA target prediction

The miRNA target prediction and analysis were performed with the algorithms from TargetScan (http://www.targetscan.org/), PicTar (http://pictar.mdc-berlin.de/) and miRanda (http://www.microrna.org/).

### Luciferase reporter assay

Part of the wild and mutated 3′UTR of NUDT1 mRNA which contained the predicted miR-485-5p targeting regions were synthesized and inserted into the pMIR-REPORT plasmid. The β-galactosidase expression vector was used as a transfection control. For the luciferase reporter assays, 2 mg of firefly luciferase reporter plasmid, 2 mg of β-galactosidase vector, and equal doses (200 pmol) of mimics, inhibitors, or scrambled negative control RNA were transfected into the prepared cells. At 24 h after transfection, cells were analyzed using the Luciferase Assay Kit.

### Cell transfection

GC cells were seeded in plates and performed transfection using Lipofectamine 2000 (Invitrogen, Life Technologies) according to the manufacturer’s instructions. The NUDT1 overexpressing plasmid and the control plasmid were generous gifts from Dr. Wang at the Tianjin Medical University Cancer Institute and Hospital. MiR-485-5p mimics and inhibitors, the siRNAs targeting NUDT1 along with control RNA were bought from Ribobio. Lentivirus to knockdown miR-485-5p was bought from shanghai Genechem Co., LTD. Equivalent doses (2 μg) of plasmids, or equal amounts (100 pmol) of miRNA mimics/inhibitors or siRNAs were transfected into each well. The cells were harvested at 24 h after transfection for RNA detection and at 48 h for protein analysis.

### RNA isolation and quantitative RT-PCR

Total RNA was extracted using TRIzol Reagent following the manufacturer’s protocol. The expression level of miR-485-5p was analyzed by TaqMan miRNA probes. U6 snRNA was used as an internal control for miRNA, and the mRNA levels of NUDT1 were normalized to GAPDH. After the PCRs were accomplished, the cycle threshold (CT) data were calculated using fixed threshold settings, and the mean CT value was determined from triplicate PCR reactions. A comparative CT method was used and the relative levels of target genes normalized to control were calculated with the equation 2^−ΔCT^, in which ΔCT = CT_gene_ − CT_control_.

### Protein extraction and western blotting

Cells were lysed in RIPA buffer. The total proteins were separated on SDS-PAGE gels and transferred to PVDF membrane. The membrane was blocked within 5% fat-free dried milk for 1 h and then incubated overnight at 4 °C with primary anti-NUDT1 (1:2000, Abcam, ab197028), or anti-GAPDH (1:5000, Santa Cruz, sc-293335), respectively. After incubation with secondary antibodies, the protein bands were visualized.

### Cell proliferation assay

The proliferative ability of GC cells was determined by EdU proliferation assay, CCK8 assay and colony formation assay. For EdU proliferation assay, at 24 h after transfection, cells were incubated in 50 μM EdU for 5 h, and then fixed within 4% paraformaldehyde for 30 min. After treatment with 0.5% Triton X-100 for 10 min, cells were incubated in darkness with Apollo staining solution for 30 min, and nuclei were then stained with DAPI for another 30 min. For CCK8 assay, GC cells were collected at 12, 24, 36 and 48 h post-transfection, and 10 μL of CCK8 was added and incubated for 4 h. Absorbance was measured at a wavelength of 450 nm. For colony formation assay, transfected cells (500 cells per well) were seeded into 12-well plate, then fixed and stained after 7–10 days.

### Cell migration assay

Wound healing assay and transwell migration assay were used to evaluate the motility of GC cells after transfection. When the cells attached, a wound healing assay was performed. Each well was scraped with a 10 μL pipette tip to create a linear region devoid of cells. The remaining cells were cultured in the medium with 1% serum. The wound closure was monitored at 0, 6, 12, 18, and 24 h after scraping. For transwell migration assay, 24-well chambers with 8-μm pore size polycarbonate membrane were used. Transfected cells (10^5^ cells per well) were seeded into the upper chamber with 200 μL serum-free medium, and 600 μL complete medium containing 10% serum was added to the lower chamber as a chemo-attractant. After 24 h of incubation, nonmigratory cells on the upper chamber were removed slightly by cotton swabs, and the membranes were fixed with methanol and subsequently stained.

### Immunofluorescence

At 48 h after transfection, cells were fixed within 4% paraformaldehyde for 30 min. After treatment with 0.5% Triton X-100 for 10 min, cells were incubated overnight at 4 °C with primary anti-8-oxo-dG (1:600, Abcam, ab62623). After incubation with appropriate secondary antibodies, nuclei were stained with DAPI.

### Immunohistochemistry assays

All sections were deparaffinized twice with xylene and rehydrated in a graded series of ethanol. The sections were performed heat mediated antigen retrieval with Tris/EDTA buffer, and incubated overnight at 4 °C with anti-NUDT1 antibody (1:100, Abcam, ab197028). The next day, the slides were incubated with second antibodies for 40 min at 37 °C and stained with the DAB system, then counterstained with hematoxylin, dehydrated, and coverslipped. Based on the degree of cell staining and the percentage of positive cells, the expression status of NUDT1 was quantified. The specimen was scored as 0, 1, 2 or 3 for no staining, light yellow staining, brown yellow staining or dark brown staining, respectively. According to the percentage of positive cells, ≤ 10, 11–25, 26–50 or ≥ 51% was recorded as the score of 0, 1, 2 or 3, respectively. Then the two scores multiplied to be the immunohistochemical staining index. The index of less than 4 was represented of the negative expression of NUDT1. Conversely, it was considered as the positive expression of NUDT1.

### Statistical analyses

All statistical analyses were performed using IBM SPSS Statistics, Version 20.0. And all data were representative of at least three independent experiments. The Student’s *t* test was used for two-group comparisons. Clinicopathological categorical variables were compared using the χ^2^ test or Fisher’s exact test. Survival curves according to NUDT1 expression were estimated with the Kaplan–Meier method and compared using the log-rank test. Differences were considered statistically significant for *P* < 0.05.

## Results

### NUDT1 is overexpressed in GC tissues and is related to the prognosis of GC patients

**Fig. 1:**
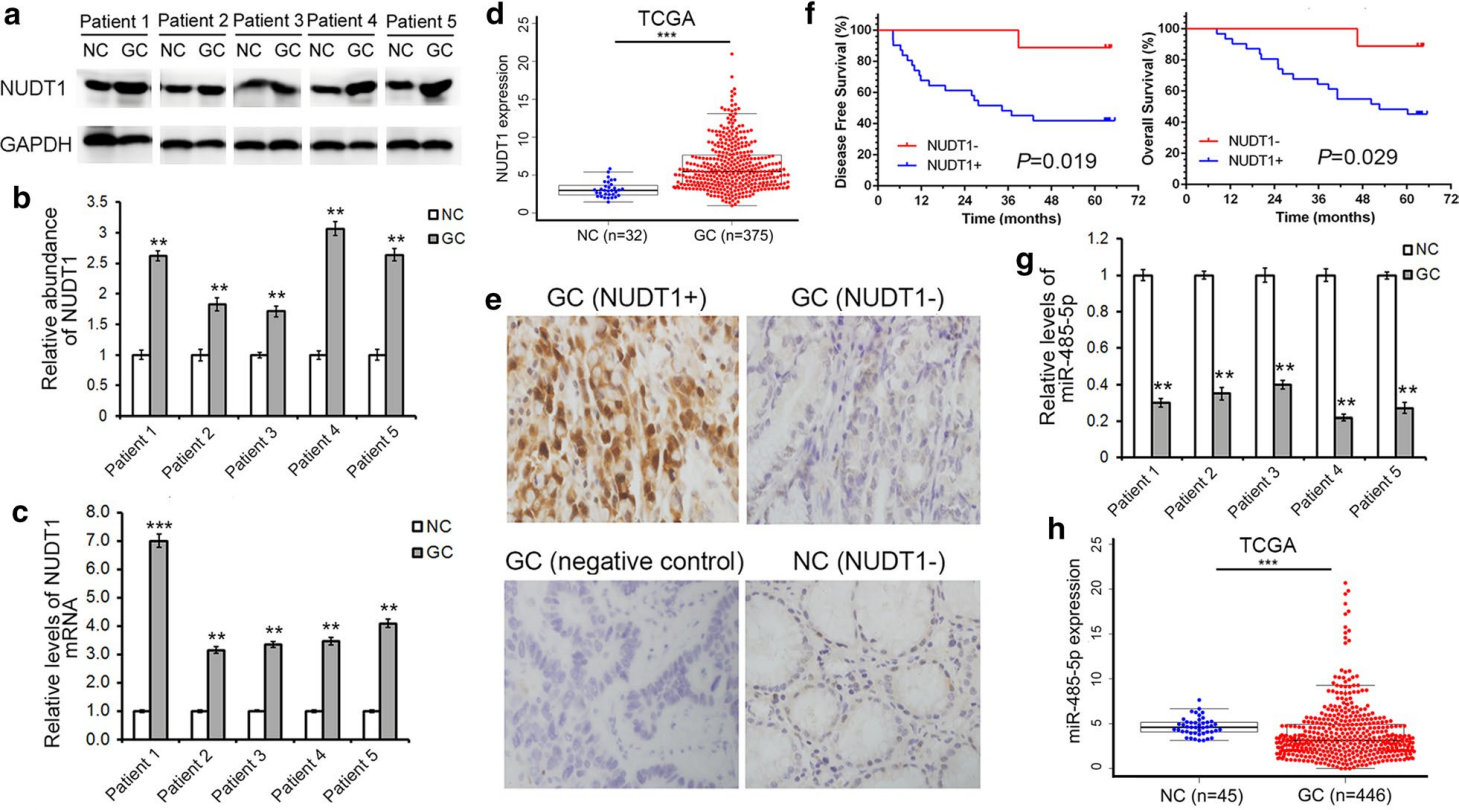
Inverse correlation between NUDT1 and MiR-485-5p in human GC tissues. **a** Western blot analysis of NUDT1 expression in GC tissues and the paired adjacent noncancerous tissues (n = 5). **b** Quantitative analysis of **a**. **c** Relative NUDT1 mRNA levels in GC tissues (n = 5). **d** NUDT1 mRNA levels in the TCGA cohort (n = 407). **e** Immunohistochemistry assays of NUDT1 expression in the paraffin-embedded GC tissues and paired noncancerous tissues (n = 40). **f** The correlation between NUDT1 expression and survival of GC patients (n = 40). **g** Relative levels of miR-485-5p in GC tissues and para-carcinoma tissues (n = 5). **h** MiR-485-5p levels in the TCGA cohort (n = 491). GC refers to gastric cancer, and NC refers to the paired non-cancerous tissue. *P < 0.05; **P < 0.01; ***P < 0.001

NUDT1 is generally known to act as a nucleotide pool sanitizing enzyme, however, its expression pattern in GC has not been detailedly described yet. In this study, the mRNA and protein levels of NUDT1 in five human GC tissues and paired noncancerous tissues were firstly evaluated. Compared with the adjacent noncancerous tissues, both the NUDT1 protein and mRNA levels were dramatically upregulated in GC tissues (Fig. 1a–c). To further investigate the expression pattern of NUDT1 in GC, a total of 407 GC patients with mRNA expression profiling were included from TCGA. As showed in Fig. 1d, the level of NUDT1 mRNA showed a clear increase in GC in accordance with our results.

Another cohort consisted of 40 gastric carcinoma patients from the Tianjin Medical University Cancer Institute and Hospital was then used to explore the expression level of NUDT1 and its relationship with clinicopathological characteristics by using IHC assays. The baseline characteristics of this cohort were listed in Table 1. NUDT1 was found to be mainly expressed in the cell cytoplasm and nucleus (Fig. 1e). Based on the staining index of each specimen, patients were divided into NUDT1-positive group and NUDT1-negative group. The expression of NUDT1 was significantly increased in the GC specimens (31/40, 77.5%), compared to the noncancerous specimens (10/40, 25.0%; χ^2^ = 22.064, *P* = 0.000). And NUDT1-positive tumors might have later T stages (*P* = 0.004). Furthermore, survival curves were constructed with the Kaplan–Meier method. The median follow-up time of this cohort was 48.9 months (range 8.3–65.5 months). The 1-, 3-, and 5-year overall survival rates were 92.5, 70.0, and 55.0% for all patients, respectively. The disease-free survival and overall survival of patients according to NUDT1 status displayed significant difference (*P* = 0.019, *P* = 0.029, respectively), and NUDT1-negative tumors tended to have favorable survival (Fig. 1f). These results showed that NUDT1 was significantly upregulated in GC, and had intense correlation with the poor prognosis of patients.

**Table 1:** The baseline characteristics of gastric carcinoma patients (n = 40)

	NUDT1-positive (n = 31) No. of patients (%)	NUDT1-negative (n = 9) No. of patients (%)	*P* value
Gender			1.000
Male	22 (71.0)	7 (77.8)	
Female	9 (29.0)	2 (22.2)	
Age			1.000
≤ 65	26 (83.9)	7 (77.8)	
> 65	5 (16.1)	2 (22.2)	
Borrmann			1.000
Type I	1 (3.2)	0 (0.0)	
Type II	17 (54.9)	5 (55.6)	
Type III	12 (38.7)	4 (44.4)	
Type IV	1 (3.2)	0 (0.0)	
Differentiation			0.643
Well/moderately	8 (25.8)	1 (11.1)	
Poorly	23 (74.2)	8 (88.9)	
T stage			0.004
T1a/T1b	0 (0.0)	2 (22.2)	
T2	1 (3.2)	2 (22.2)	
T3	1 (3.2)	1 (11.1)	
T4a	29 (93.6)	4 (44.5)	
N stage			0.157
N0	12 (38.7)	2 (22.2)	
N1	2 (6.5)	3 (33.3)	
N2	8 (25.8)	3 (33.3)	
N3a/N3b	9 (29.0)	1 (11.2)	

### Identification of miR-485-5p as a potential upstream regulator of NUDT1

**Fig. 2:**
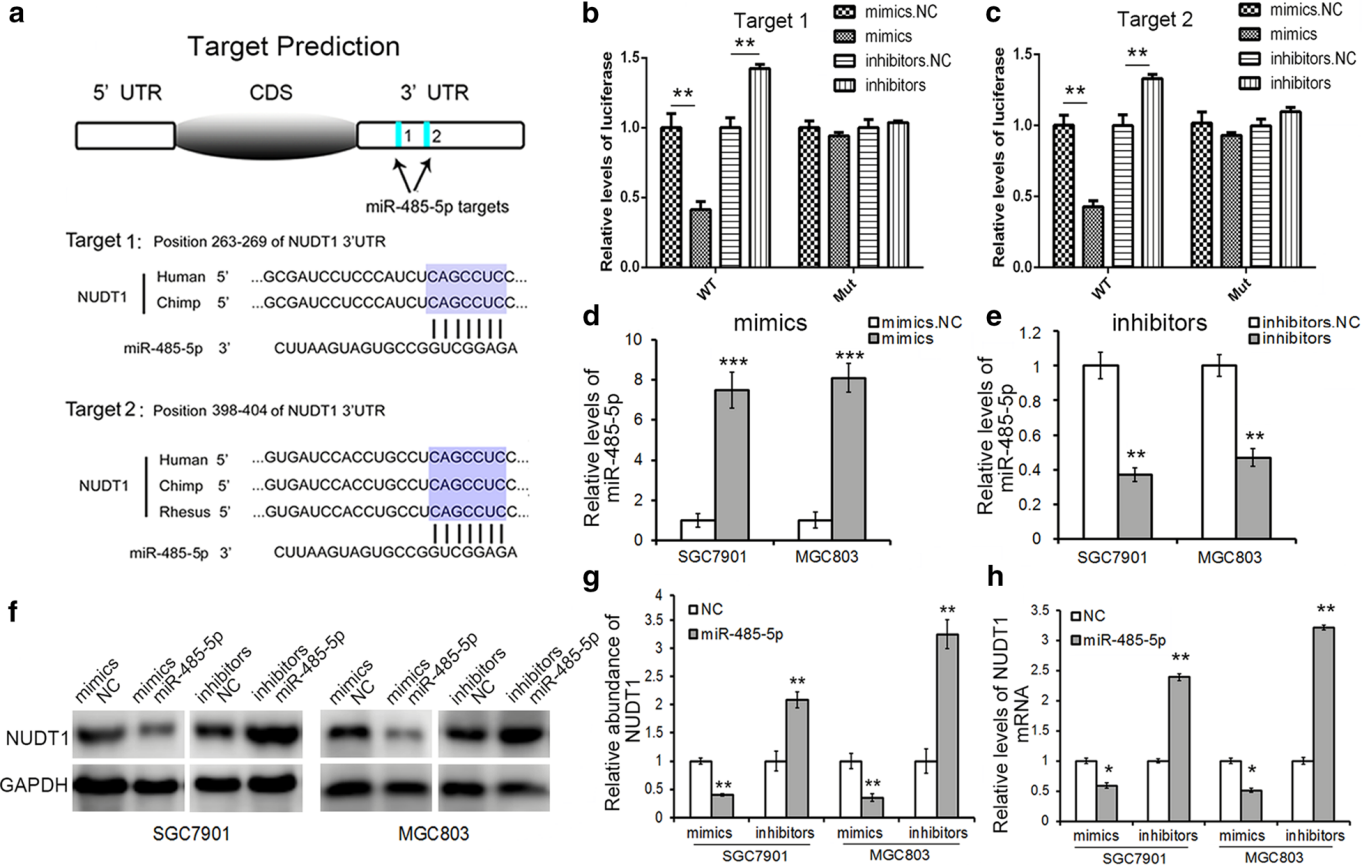
Validation of NUDT1 as a direct target of miR-485-5p in GC cells. **a** The predicted binding sites and base-pairing interaction between miR-485-5p and the 3′UTR of NUDT1 mRNA. **b**, **c** Direct recognition of NUDT1 by miR-485-5p. HEK-293T cells were co-transfected with firefly luciferase reporters containing either WT or mutant NUDT1 3′UTR with miR-485-5p mimics or inhibitors. **d**, **e** Quantitative RT-PCR analysis of miR-485-5p levels in GC cells transfected with miR-485-5p mimics (**d**) or inhibitors (**e**). **f** The regulation of NUDT1 expression by miR-485-5p in GC cells. **g** Quantitative analysis of **f**. **h** Quantitative RT-PCR analysis of NUDT1 mRNA levels in GC cells transfected with miR-485-5p mimics or inhibitors. *P < 0.05; **P < 0.01; ***P < 0.001

Through specific degradation or translational repression of target mRNA, miRNAs play critical roles in regulating the expression of oncogenes and tumor suppressors. Whether miRNAs can induce the dys-regulation of NUDT1 in GC has not been investigated yet. In the present study, TargetScan, PicTar and miRanda were used to predict the probable upstream regulator of NUDT1. The results displayed that miR-485-5p could directly bind to the 3′UTR of NUDT1 mRNA by complementary base pairing of two target regions, as showed in Fig. 2a, the binding sites were highly conserved.

It was reported that miR-485-5p was downregulated in various cancers, including GC. The expression pattern of miR-485-5p in the five pairs of GC tissues and corresponding noncancerous tissues was then evaluated. As expected, the expression level of miR-485-5p was significantly decreased in all the GC tissues (Fig. 1g). Similarly, miR-485-5p was found to be down-regulated in the TCGA cohort (Fig. 1h). The levels of miR-485-5p and NUDT1 showed inverse correlation both in our cohort and TCGA cohort, thus miR-485-5p was speculated to be a potential regulator of NUDT1 in GC.

### Validation of NUDT1 as a direct target of miR-485-5p in GC cells

Luciferase assays were performed in HEK-293T cells to verify the direct interaction between miR-485-5p and NUDT1. As showed in Fig. 2b, c, the luciferase signal was significantly decreased by nearly 50% when miR-485-5p mimics were co-transfected with the wild-type luciferase reporters, however, the inhibition was lost when the binding sites in 3′UTR were mutated instead. The relative luciferase activity showed obvious increase when miR-485-5p inhibitors were co-transfected. And the results of the two predicted targets were consistent.

Then, the regulatory effects of miR-485-5p on NUDT1 expression were evaluated in GC cells. The expression levels of miR-485-5p in GC cell lines were reported to be downregulated compared to the normal gastric cell line (GES-1). SGC7901 cell line and MGC803 cell line were selected for further experiments in this study. MiR-485-5p mimics or inhibitors were utilized to achieve the up-regulation or down-regulation of miR-485-5p (Fig. 2d, e), respectively. Overexpression of miR-485-5p led to a sharp reduction of NUDT1 mRNA and protein, whereas the inhibition of miR-485-5p clearly enhanced the expression of NUDT1 (Fig. 2f–h). To further validate the role of miR-485-5p, lentivirus to knockdown its expression was used. As Additional file 1: Figure S1 showed, knockdown of miR-485-5p lead to the overexpression of NUDT1 at both protein and mRNA levels. The above results indicated that miR-485-5p could directly target NUDT1 mRNA and induce its degradation.

### Suppressive role of miR-485-5p in GC cells

**Fig. 3:**
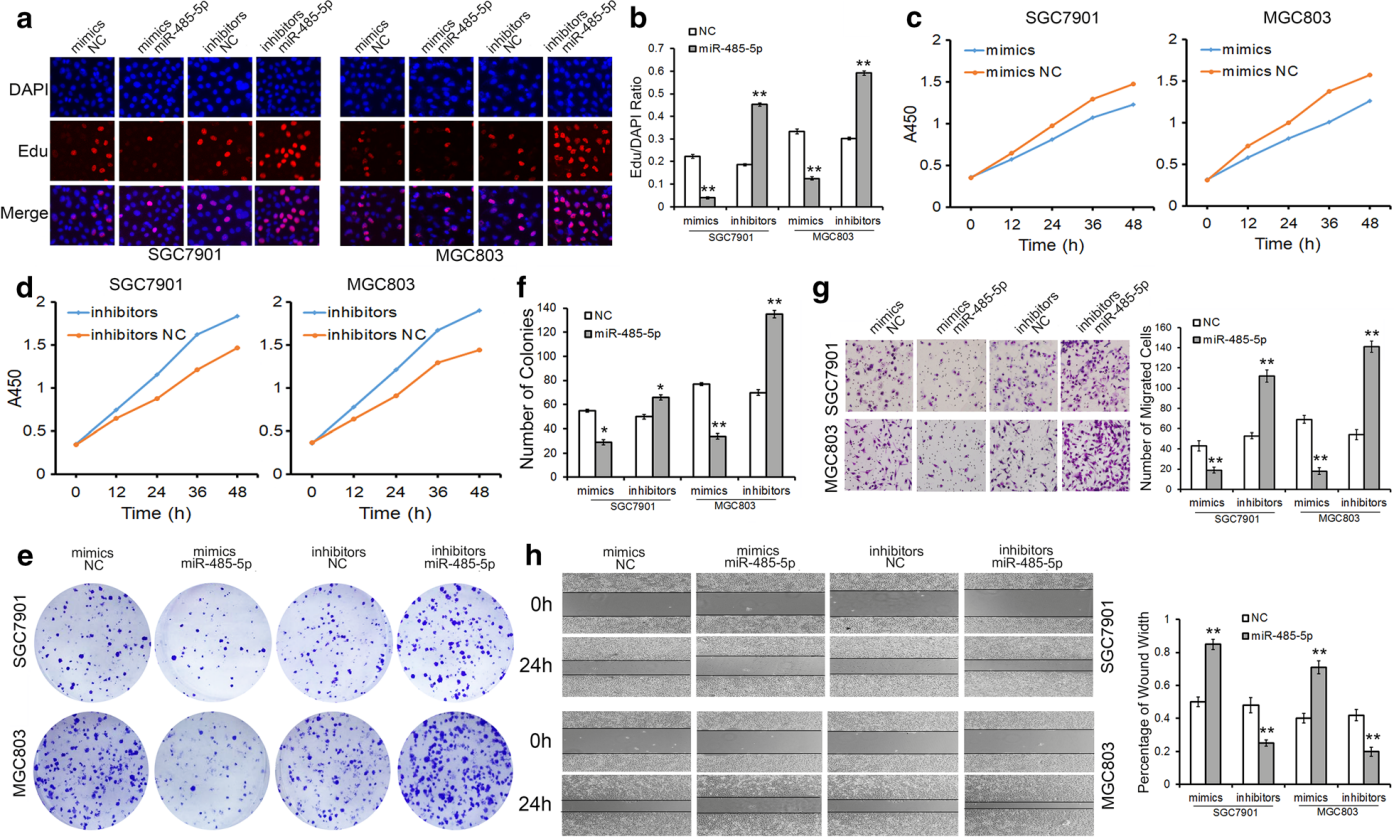
MiR-485-5p suppresses the proliferation and migration of GC cells. **a**–**f** EdU assays (**a**, **b**), CCK8 assays (**c**, **d**) and colony formation assays (**e**, **f**) demonstrate that the overexpression of miR-485-5p inhibits the proliferation of SGC7901 and MGC803 cells. **g**, **h** Transwell assays (**g**) and wound healing assays (**h**) demonstrate that the overexpression of miR-485-5p suppresses the migration of GC cells. **P < 0.01

We further assessed cell growth and cell motility in GC cells transfected with miR-485-5p mimics/inhibitors. The EdU proliferation assay, CCK8 assay and colony formation assay were used to investigate the proliferation of GC cells. As expected, the overexpression of miR-485-5p resulted in a sharp decrease of cell proliferation, whereas knock-down of miR-485-5p led to increasing cell proliferation significantly (Fig. 3a–f). Effects of miR-485-5p on cell migration were evaluated by transwell assay (Fig. 3g) and wound healing assay (Fig. 3h). Both the two approaches demonstrated that GC cells transfected with miR-485-5p inhibitors showed a higher ratio in migration, whereas cell migration was strongly inhibited when cells are transfected with miR-485-5p mimics. These results suggest that miR-485-5p acts as a tumor suppressor in GC and its dramatic down-regulation contributes to a faster rate of proliferation and increased migration in cancer cells.

### NUDT1 promotes cell proliferation and migration in GC cells

**Fig. 4:**
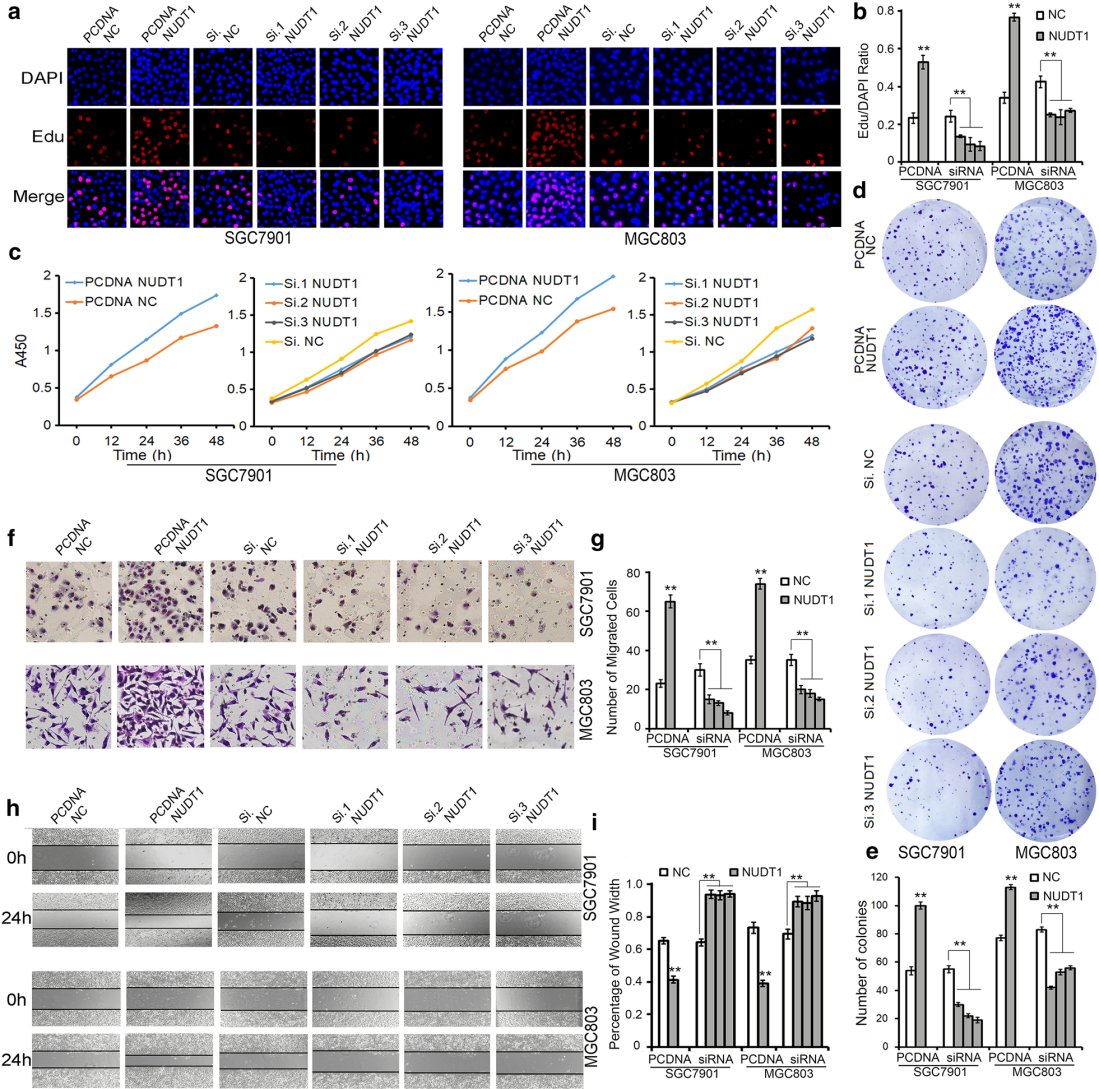
Identification of NUDT1 as an oncogene in GC. **a**–**e** EdU assays (**a**, **b**), CCK8 assays (**c**) and colony formation assays (**d**, **e**) demonstrate that up-regulation of NUDT1 promotes cell proliferation of GC cells. **f**–**i** Transwell assays (**f**, **g**) and wound healing assays (**h**, **i**) show that overexpression of NUDT1 enhances cell migration of GC cells strongly. PCDNA NUDT1 refers to overexpression plasmid, and PCDNA NC refers to control plasmid. **P < 0.01

To give a better understanding of NUDT1-involved pathway in GC, plasmid or siRNAs were used to overexpress or knock-down NUDT1 in GC cells, respectively. The silencing and overexpression efficiency was assessed by RT-qPCR and western blotting. Both the mRNA and protein levels of NUDT1 were augmented by the overexpression plasmid (Additional file 2: Figure S2A–C) and were markedly inhibited by siRNAs (Additional file 2: Figure S2D–F). The function of NUDT1 on cell growth and migration was measured subsequently. The proliferation assay (Fig. 4a–e) indicated that the proliferation rate of GC cells overexpressing NUDT1 was significantly higher than that of control cells, whereas siRNAs targeting NUDT1 could inhibit cell proliferation dramatically. Furthermore, cells with upregulated NUDT1 showed stronger ability to migrate, while knock-down of NUDT1 could inhibit cell migration significantly (Fig. 4f–i). Therefore, as an oncogene of GC, NUDT1 promotes cell proliferation and migration obviously.

### The impacts of miR-485-5p/NUDT1 axis on eradicating 8-oxo-dG in GC cells

**Fig. 5:**
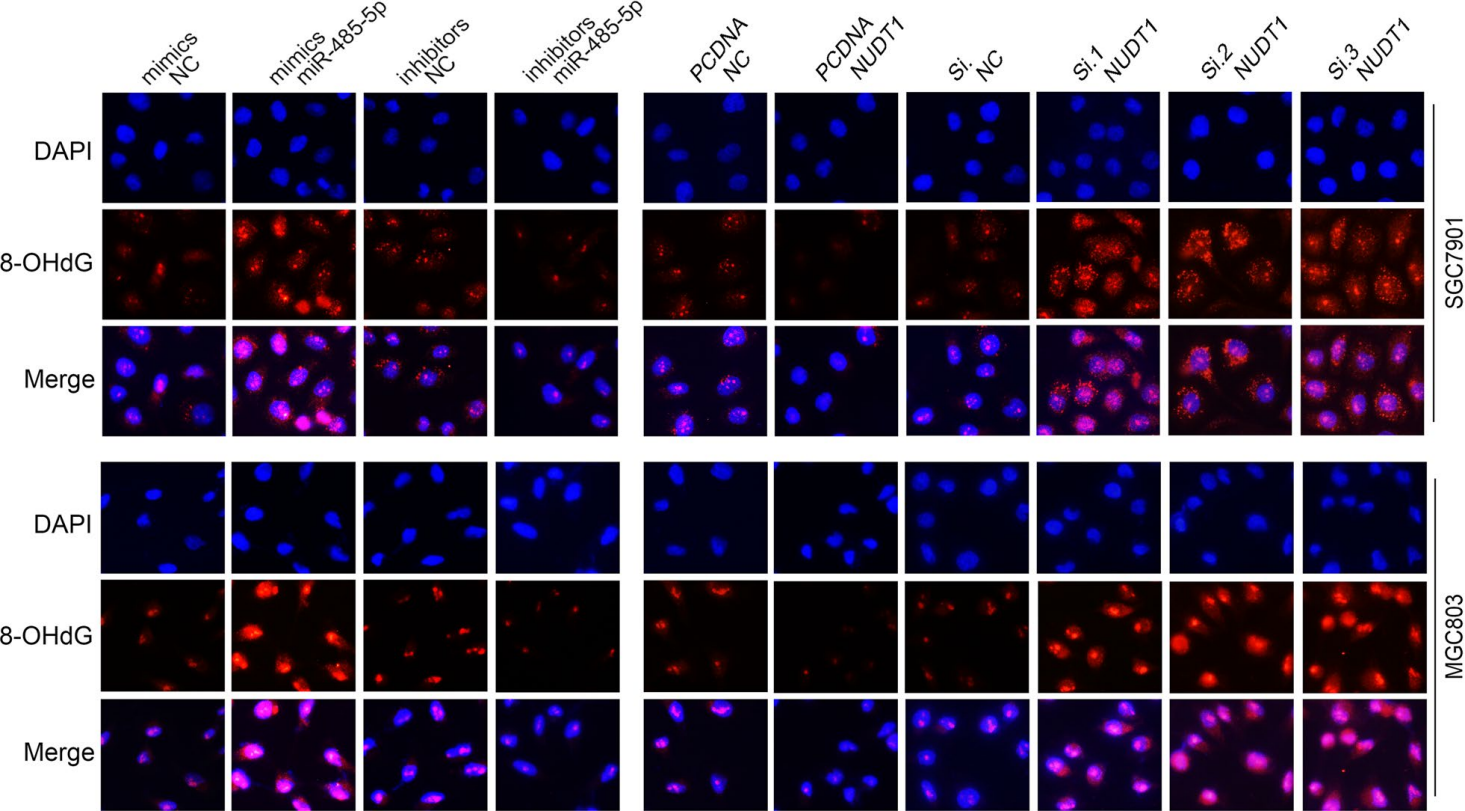
The impacts of miR-485-5p/NUDT1 axis on eradicating 8-oxo-dG in GC cells. The 8-oxo-dG in the nucleotide pools was detected by immunofluorescence. When NUDT1 was up-regulated by plasmid or miR-485-5p inhibitors, the 8-oxo-dG in nucleus and cytoplasm decreased; while the level of 8-oxo-dG increased after the knockdown of NUDT1. PCDNA NUDT1 refers to overexpression plasmid, and PCDNA NC refers to control plasmid

NUDT1 was known as an 8-oxo-dGTPase, hence its role in eradicating 8-oxo-dG was investigated. Immunofluorescence was used to detect the cellular level of 8-oxo-dG in the nucleotide pools of GC cells after transfection. As showed in Fig. 5, when NUDT1 was up-regulated by plasmid or miR-485-5p inhibitors, the 8-oxo-dG in nucleus and cytoplasm decreased; whereas the level of 8-oxo-dG increased after the knockdown of NUDT1. The miR-485-5p/NUDT1 axis could lead to the changes of 8-oxo-dG, thus contributing to malignant phenotypes.

### The effects of miR-485-5p/NUDT1 axis on GES-1 cells

**Fig. 6:**
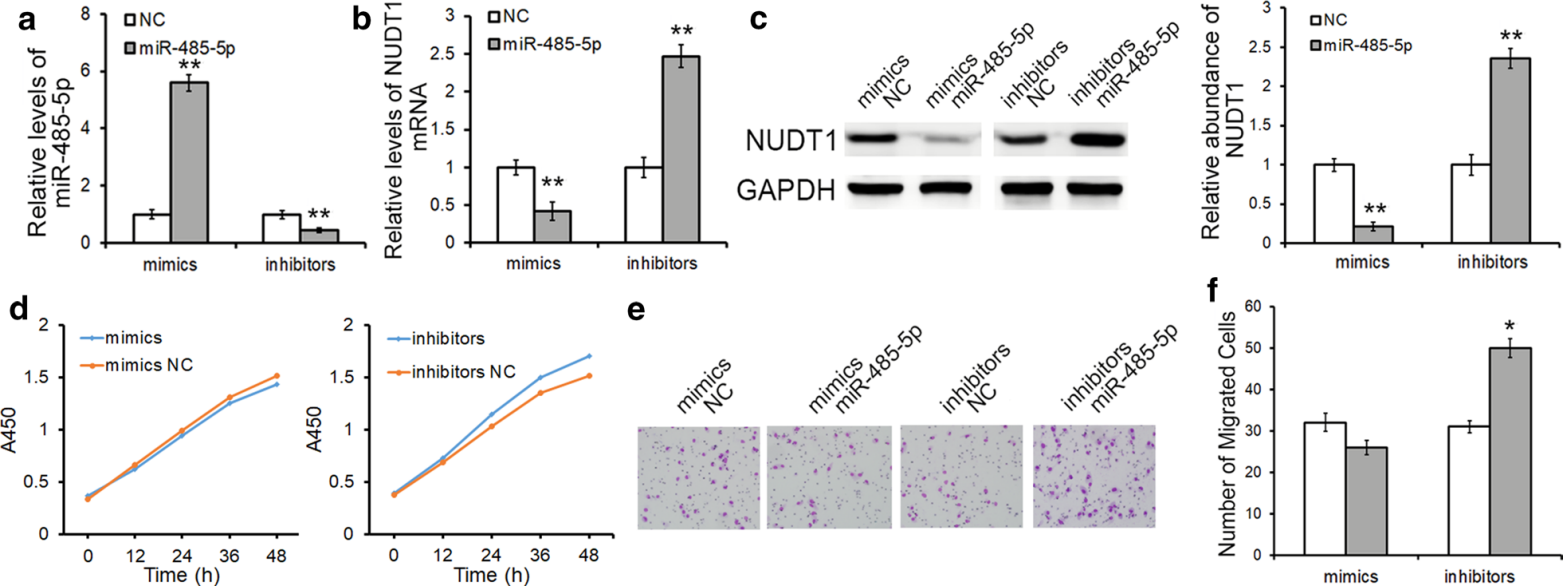
The effects of miR-485-5p/NUDT1 axis on normal gastric cells. **a** Quantitative RT-PCR analysis of miR-485-5p levels in GES-1 cells transfected with miR-485-5p mimics or inhibitors. **b** Relative NUDT1 mRNA levels after transfection. **c** The regulation of NUDT1 expression by miR-485-5p. **d**–**f** The up-regulation of miR-485-5p had no effect on the proliferation (**d**) and migration (**e**, **f**) of GES-1 cells, whereas the knockdown of miR-485-5p could promote malignant phenotype. *P < 0.05; **P < 0.01

The results above showed that siRNA-mediated NUDT1 down-regulation could simulate the effects of miR-485-5p mimics on phenotypes of GC cells, while plasmid-mediated NUDT1 overexpression had the identical effects with miR-485-5p inhibitors. To further validate the role of miR-485-5p/NUDT1 axis in GC, normal gastric cell line (GES-1) was used as a control. Although miR-485-5p mimics lead to a sharp decrease of NUDT1 in GES-1 cells (Fig. 6a–c), it had no effect on the proliferation and migration of GES-1 cells (Fig. 6d–f), which demonstrated that the growth of normal cells is not dependent on the protection of NUDT1. However, the overexpression of NUDT1 could partially accelerate the proliferation and migration in GES-1 cells (Fig. 6d–f), suggesting the oncogenic role of NUDT1.

## Discussion

In our present study, miR-485-5p and NUDT1 levels showed opposite trends in 5 pairs of gastric carcinoma and noncancerous tissues. The overexpression of NUDT1 was related to later T stage and indicated poorer survival in GC patients. MiR-485-5p was verified to be an upstream regulator of NUDT1, and the dramatic knock-down of miR-485-5p in GC leads to upregulation of NUDT1, thus contributing to accelerated proliferation and increased migration of cancer cells.

NUDT1, acts as a nucleotide pool sanitizing enzyme, plays an indispensable part in surviving the oxidative stress in cancer cells. However, several researchers found distinct roles of NUDT1 [25, 26], in which NUDT1 deficiency in certain cancer cell lines achieved by small RNA interference or genome editing does not result in any detrimental effects on these cells, indicating that NUDT1 may not be always indispensable for cancer cell survival under oxidative conditions. Hence, the authentic role of NUDT1 in oncogenesis needs more investigation. In accordance with the results of most previous studies, we found that the expression of NUDT1 was up-regulated at both mRNA and protein levels in GC. The oncogenic role of NUDT1 which we verified in the GC cells and GES-1 cells can provide another proof for the therapy targeting NUDT1. By far, some NUDT1 inhibitors have been discovered and have showed some favorable effects in vitro and in vivo [16, 27–29].

Although miR-485-5p has been found to be down-regulated in both GC tissues and cell lines [23, 24], its specific mechanism in the process of tumorigenesis remains unclear. We identified that miR-485-5p could directly bind to the 3′UTR of NUDT1 mRNA, then lead to its clearance in GC. MiRNA-mediated repression or degradation of mRNA transcripts is one of the essential modes of post-transcriptional regulation. It has been under investigated how the miRNA-mRNA interaction decides whether to degrade the mRNA or repress its translation. Researchers concluded that the short length of 3′UTR [30] or the A/U-rich 3′UTRs [31] were positively associated with the degradation of mRNA. The 3′UTR of NUDT1 mRNA is short, and it may be one reason why miR-485-5p can lead to the clearance of NUDT1 mRNA. The miRNA-NUDT1 pathway has been investigated previously in lung cancer [32]. It was pointed out that miR-145 could inhibit cell proliferation and was in the negative regulation of NUDT1 expressions at both mRNA and protein levels [32]. Since one miRNA can target multiple genes and one gene can be regulated by several miRNAs, identification of the complete miRNA-gene pathway is necessary and can provide more insights into the exploration of new therapeutic targets. The inhibitory effects of miR-485-5p on GC cells can clue to the treatment based on miR-485-5p.

It is the first time to detailedly describe the expression pattern and biological role of NUDT1 in GC. And the correlation between NUDT1 expression and survival of GC patients has not been identified previously. The miR-485-5p/NUDT1 axis is verified to be involved in the carcinogenesis of GC, however, additional cohorts and in vivo experiments are needed.

## Conclusions

To conclude, experimental evidence in our study proves that miR-485-5p possesses a tumor-suppressive function in GC and the down-regulated miR-485-5p lead to the increased expression of NUDT1, thus contributing to cancer progression. MiR-485-5p/NUDT1 axis is involved in the processes of cell growth and cell motility and plays key roles in the tumorigenesis of GC. Our findings provide evidence for the function of miR-485-5p in GC and can clue to the treatment by targeting NUDT1 in cancer.

## Additional files

**Additional file 1: Figure S1.** The role of miR-485-5p knockdown lentivirus. A. Quantitative RT-PCR analysis of miR-485-5p levels in GC cells. B. Relative NUDT1 mRNA levels. C. The regulation of NUDT1 expression by miR-485-5p knockdown lentivirus. D. Quantitative analysis of C. ** indicates *P* < 0.01.

**Additional file 2: Figure S2.** Overexpression and knock-down of NUDT1 in GC cells. A, B and C. Up-regulation of NUDT1 expression by plasmid. GC cells were transfected with NUDT1 plasmid, and the protein levels (A and B) and mRNA levels (C) were detected respectively. D, E and F. Silencing of NUDT1 expression by siRNAs. GC cells were transfected with NUDT1 siRNAs, and the protein levels (D and E) and mRNA levels (F) were detected respectively. PCDNA NUDT1 refers to overexpression plasmid, and PCDNA NC refers to control plasmid. ** indicates *P* < 0.01; *** indicated *P* < 0.001.

## Abbreviations

miRNAs: microRNAs
GC: gastric cancer
NUDT1: nucleoside diphosphate linked moiety X-type motif 1
MTH1: MutT homolog-1
ROS: reactive oxygen species
TCGA: The Cancer Genome Atlas
