# Supplementary figures and images for: The role of miR-485-5p/NUDT1 axis in gastric cancer
Source: Cancer Cell Int. 2017 Oct 17;17:92. doi: 10.1186/s12935-017-0462-2 (PMC5645910; doi:10.1186/s12935-017-0462-2)

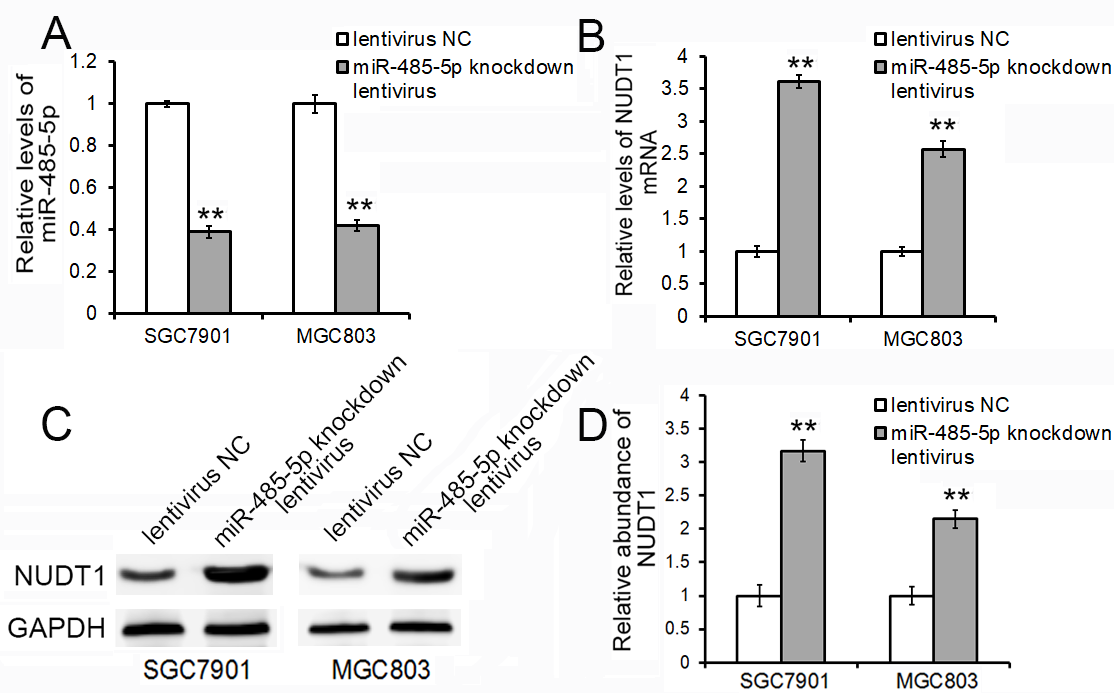

Supplement: Supplementary file 1 — Additional file 1: Figure S1. The role of miR-485-5p knockdown lentivirus. A. Quantitative RT-PCR analysis of miR-485-5p levels in GC cells. B. Relative NUDT1 mRNA levels. C. The regulation of NUDT1 expression by miR-485-5p knockdown lentivirus. D. Quantitative analysis of C. ** indicates P < 0.01. [file 12935_2017_462_MOESM1_ESM.tif]

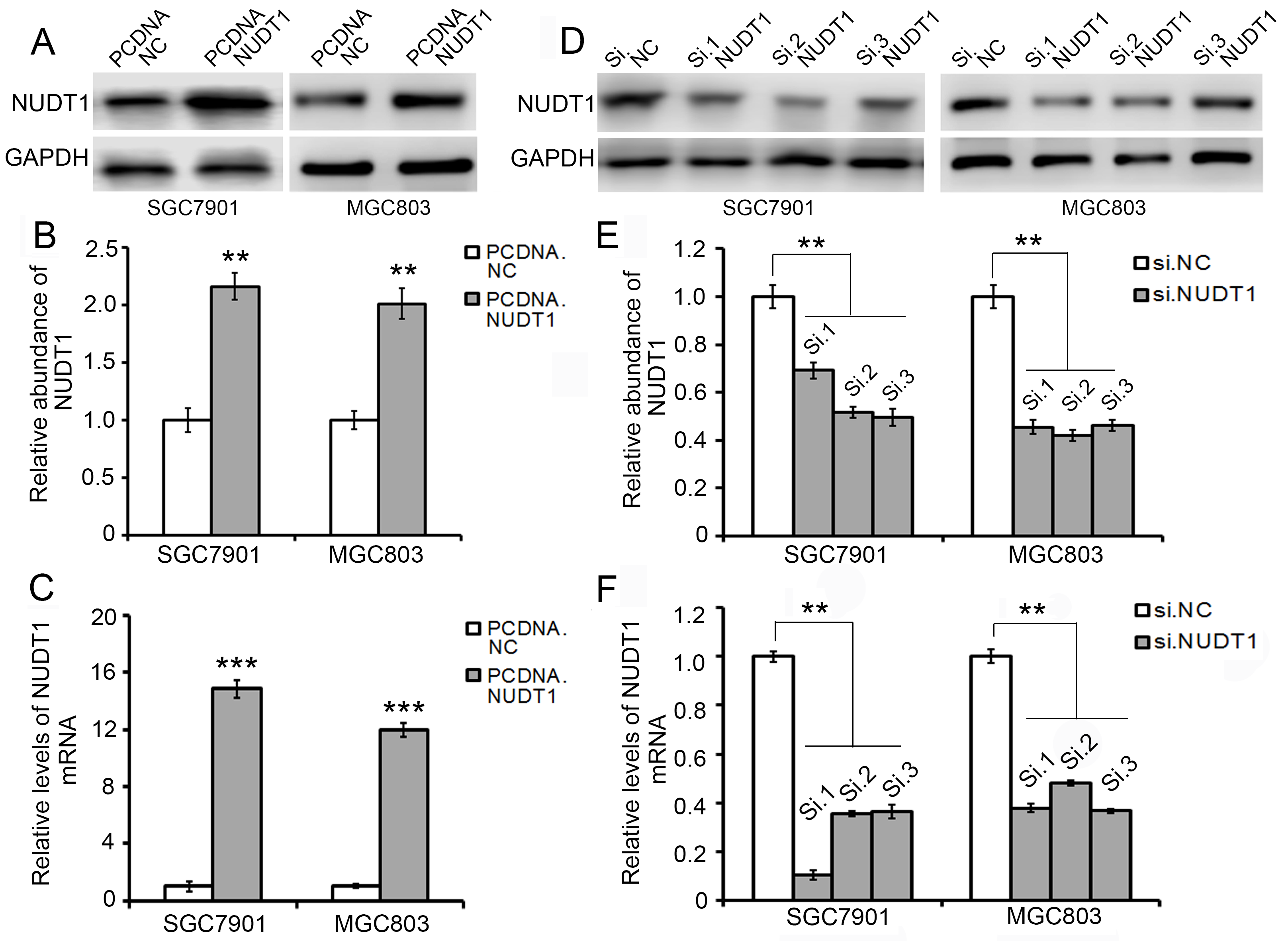

Supplement: Supplementary file 2 — Additional file 2: Figure S2. Overexpression and knock-down of NUDT1 in GC cells. A, B and C. Up-regulation of NUDT1 expression by plasmid. GC cells were transfected with NUDT1 plasmid, and the protein levels (A and B) and mRNA levels (C) were detected respectively. D, E and F. Silencing of NUDT1 expression by siRNAs. GC cells were transfected with NUDT1 siRNAs, and the protein levels (D and E) and mRNA levels (F) were detected respectively. PCDNA NUDT1 refers to overexpression plasmid, and PCDNA NC refers to control plasmid. ** indicates P < 0.01; *** indicated P < 0.001. [file 12935_2017_462_MOESM2_ESM.tif]
